# Isolated bilateral pleural effusion as the sole manifestation of late onset ovarian hyperstimulation syndrome

**DOI:** 10.4103/0974-1208.57229

**Published:** 2009

**Authors:** Shalu Gupta, Balasubramaniam Sathya, Nabaneeta Padhy, Shankar Kundavi, Betty E Thomas, Thangam R Varma

**Affiliations:** 1 Department of Obstetric and Gynaecology, Reproductive Medicine, Institute of Reproductive Medicine, Madras Medical Mission, J J Nagar, Chennai, Tamil Nadu, India; 2 Repro Medicine, Institute of Reproductive Medicine, Madras Medical Mission, J J Nagar, Chennai, Tamil Nadu, India; 3 Department of Obstetric and Gynaecology, Reproductive Medicine, Institute of Reproductive Medicine, Madras Medical Mission, J J Nagar, Chennai, Tamil Nadu, India; 4 MNAMS, Institute of Reproductive Medicine, Madras Medical Mission, J J Nagar, Chennai, Tamil Nadu, India; 5 Repro Medicine, Institute of Reproductive Medicine, Madras Medical Mission, J J Nagar, Chennai, Tamil Nadu, India; 6 FRCOG, Institute of Reproductive Medicine, Madras Medical Mission, J J Nagar, Chennai, Tamil Nadu, India

**Keywords:** Isolated pleural effusion, ovarian hyperstimulation syndrome, late onset

## Abstract

To report a case of late onset ovarian hyperstimulation with bilateral pleural effusion and respiratory distress as the sole manifestation after embryo transfer.

## INTRODUCTION

Ovarian hyperstimulation syndrome (OHSS) is the most grave complication of controlled ovarian hyperstimulation. It is second only to high-order pregnancy on the list of adverse outcomes that need to be minimized or eliminated. Pulmonary manifestations were observed in 7.2% of severe OHSS cases.[1]

This article reports an unusual case of isolated bilateral pleural effusion after ovarian stimulation and *in vitro* fertilization (IVF) with a review of the literature.

## CASE REPORT

A 21 year-old female married at the age of 18 years attended the Institute of Reproductive Medicine out patient clinic with secondary subfertility of 2 years, irregular cycles and polycystic ovaries.

The patient had already undergone five cycles of ovulation induction (clomiphene citrate and gonadotropin) with intra-uterine insemination (IUI) and laparoscopy showing bilateral sacculation of tubes with slow spill at both ends.

Obstetric history included missed abortion at 8 weeks of gestation, 2 years back.

There was no past history of chronic pulmonary obstructive disease, asthma or tuberculosis.

Also, there was no family history of any chronic illness, including thrombo-embolism.

Clinical examination showed a body mass index of 24 kg/m^2^ and no evidence of hyperandrogenism.

Pelvic ultrasound scan revealed polycystic ovaries and a normal-sized anteverted uterus. A baseline hormonal profile performed on day 2 of the menstrual cycle showed a follicle- stimulating hormone (FSH) concentration of 4 miu/ml, luteinizing hormone concentration of 6 miu/ ml and an estradiol concentration of 32 pg/ ml. The total antral follicle count was 24, with a right ovary volume of 11 cc and a left ovary volume of 12 cc. The husband's semen analysis showed normozoospermia.

The patient was planned for ovulation induction with IUI, explaining the risk of hyperstimulation and thereafter converted to IVF. She underwent controlled ovarian stimulation according to the flexible gonadotropin-releasing hormone antagonist protocol. She was started on a low- dose injection of hp FSH 75U (Bravelle; Ferring Pharmaceuticals, [India]). The dosage was increased to 150U after 6 days of gonadotropin as there was no response. A scan done after 2 days showed recruitment of more than six follicles and, after recounselling, was converted to IVF cycle. When three follicles reached a mean diameter of ≥18 mm (folliculogram: ≥14 mm, eight follicles; 10-14 mm, three follicles; < 10 mm, four follicles), the patient received 10,000 units of urinary human chorionic gonadotropin (hCG) (Choragon; Ferring Pharmaceuticals). On that day, her estradiol was 1272 pg/ml. Ten oocytes were retrieved, of which seven fertilized. On day 3, three grade A embryos were transferred and the remaining four were cryopreserved.

The patient presented with breathlessness and orthopnea 11 days after embryo transfer (day 16 post hCG). There was no history of fever, cough or chest pain.

On examination, her pulse rate was 90/min, blood pressure 120/70 mmHg, temperature 37°C and respiratory rate 24/min. On chest auscultation, decreased breath sounds at the basal area of both the lungs with occasional crepitations were heard. The abdomen was soft, bowel sounds were present and there was no clinical evidence of ascites.

Ultrasonography (both transvaginal and transabdominal) of the abdomen and chest showed bilateral enlarged ovaries (90 × 85 mm and 85 × 54 mm) with no fluid collection in the pelvis. However, there was evidence of bilateral pleural effusion. The oxygen saturation on air measured with a pulse oximeter was 96%. Laboratory investigation revealed a hematocrit value of 34% and white blood cell count of 11,000 cells/cub.mm. βhCG was 236 miu/ml (11 days post-embryo transfer). Her renal and hepatic parameters were normal.

Echocardiography (Echo) showed good LV function with mild pericardial effusion.

Provisional diagnosis of bilateral hydrothorax secondary to late onset OHSS.

The other differentials considered were tuberculosis and pulmonary embolism.

Tuberculosis was considered because of its high prevalence in India. It was ruled out as the patient presented with short history, with no constitutional symptoms, no history of exposure or family history and routine chest X-ray performed at the time of laparoscopy was normal.

The patient's condition was stable and oxygen saturations were maintained.

She was managed conservatively with a multidisciplinary approach and intensive care monitoring. She was placed in a propped-up position along with chest physiotherapy, intermittent O_2_ inhalation and nebulization.

Echo and ultrasound scan later confirmed resolution of pericardial and pleural effusion.

Fortunately, the patient did not require thoracocentesis as her symptoms subsided. She was discharged after 1 week and repeat investigation revealed doubling of serum βhCG.

Transvaginal ultrasound performed 1 week later showed twin intrauterine gestation, which is ongoing.

## DISCUSSION

OHSS complicates almost 33% of cycles of ovarian stimulation. The incidence of severe form varies between 3 and 8% of IVF cycles[2] and can be complicated by life-threatening complications.

Of pulmonary manifestations, dyspnoea and tachypnoea are the most common symptoms. Other less common manifestations are pneumonia, adult respiratory distress syndrome, and pulmonary thromboembolism.[1] Hydrothorax (pleural effusion) is reported in about 10% of cases, which is usually associated with marked ascites.[3] However, the incidence of pleural effusion with or without ascites is more common than reported.

Routes of transfer of ascitic fluid to pleural space include (i) through diaphragmatic lymphatic as in chronic ascites and (ii) anatomical defects observed during laparoscopy and laparotomy.[4] Thin membrane covers these macroscopic defects in the tendenious portion of the diaphragm, which are converted into blebs due to high intra-abdominal pressure because of the ascitic fluid. When these blebs rupture, the negative thoracic pressure allows ascitic fluid to permeate into the pleural space.

These defects are more common in females and on the right side of the diaphragm, explaining the predominance of right sided effusion.

Frequently, small unilateral or bilateral pleural effusions are observed without pulmonary compromise, which resolve spontaneously. Isolated hydrothorax is neither adequately acknowledged in scoring tables nor is its pathogenesis clear.[5]

It is unlikely that a systemic peptide will target its vascular permeability effects on pleural spaces without affecting any other serosal surface. Furthermore, pleural fluid interleukin 6 levels are comparable to values reported in ascites, which is 1000 times higher than normal serum, also supporting a passive movement of ascitic fluid from the abdomen into the pleural space.[6]

Literature revealed 30 cases of isolated hydrothorax as the sole manifestation of OHSS.[5,7–23]

In almost all these cases, the hydrothorax was part of late onset type of OHSS, although it could manifest as early OHSS also.[5,24]

Risk factors identified by reviewing the literature –

- young age (mean, 30 years; range, 24-38)
- presence of polycystic ovarian syndrome (PCOS) in 25% of the cases
- large number of oocytes retrieved (mean, 17 oocytes; range, 10-26) and
- ongoing pregnancy in 60% of the cases.

But, hydrothorax can occur in older and low-risk patients also.[24]

Prompt initiation of thoracocentesis[21,25] and multidisciplinary approach have been found to have favorable prognosis.

Biochemical parameters, hemoconcentration and leukocytosis are useful in differentiating hydrothorax associated with OHSS from other chest conditions such as pulmonary embolism.[24]

Our case is unusual as the patient in spite of low-dose stimulation, peak estradiol level of 1272 pg/ml on day of hCG and with as few as 10 oocytes retrieved, developed bilateral pleural effusion after ovarian stimulation.

It also shows that late-onset OHSS can present as isolated pleural effusion without hemoconcentration and leukocytosis.

In women who present with a history of dyspnoea following the use of exogenous gonadotropins, a detailed evaluation of the respiratory system is warranted, including ultrasonography of the chest apart from clinical examination. In case pleural effusion is found, its cause is most likely to be secondary to controlled ovarian hyperstimulation (even in the absence of other features suggestive of OHSS and normal laboratory parameters).

Imaging and assessment of the chest are necessary to diagnose the degree of pulmonary compromise and active intervention as and when needed.

## CONCLUSIONS

The frequency of OHSS presenting with an isolated pleural effusion is probably underestimated in the literature due to its spontaneous favorable outcome. It can present without the usual risks of OHSS, i.e. young age, PCOS, hyperstimulation or hemoconcentration, with a wide range of clinical scenario ranging from massive ascites to no ascites with normal biochemical parameters. In any patient diagnosed to have pleural effusion following controlled ovarian stimulation, OHSS should be considered as the most likely cause. Gynecologists and chest physicians should be more aware of this syndrome in order to ensure timely diagnosis and better management of these potentially pregnant patients.
